# Caught on Camera: Insights Into Mizoram's Mammalian Diversity Through a Camera‐Trap‐Based Distance Sampling Approach

**DOI:** 10.1002/ece3.72501

**Published:** 2025-12-05

**Authors:** Akangkshya Priya Gogoi, Joonu Chakma, Lallianpuii Kawlni, Vishnupriya Kolipakam, Qamar Qureshi

**Affiliations:** ^1^ Wildlife Institute of India Dehradun Uttarakhand India; ^2^ World Wildlife Fund for Nature‐India New Delhi India

**Keywords:** density, hunting, hurdle modelling, relative abundance index, temporal activity

## Abstract

Assessing species distribution and associated threats is crucial for effective conservation. Many species including mammals face extinction due to habitat loss, hunting, and illegal trade, with their populations largely confined to protected areas. Mizoram, situated within the Indo‐Myanmar biodiversity hotspot, is increasingly threatened by illegal wildlife trade and lacks comprehensive faunal studies, necessitating urgent scientific research and conservation interventions. We conducted a camera‐trap‐based density estimation in selected protected areas of Mizoram to understand the population status of mammal species, evaluate the density of ungulates, and the relative abundance of major predator and ungulate species. Barking deer (*Muntiacus vaginalis*), sambar ( *Rusa unicolor* ), wild pig ( *Sus scrofa* ), red serow ( *Capricornis rubidus* ), and gaur ( *Bos gaurus* ) were the major ungulates photo‐captured from the region. Clouded leopards ( *Neofelis nebulosa* ) and dhole ( *Cuon alpinus* ) were the major predators detected from camera trapping. The relative abundance of clouded leopard (1.82 ± 0.93) and dhole (1.73 ± 1.10) was highest in Dampa Tiger Reserve relative to other protected areas of northeast India. However, the relative abundances and densities of ungulates were noticeably lower relative to the estimates from other protected areas in India. Illegal hunting may be one of the causes of low species abundance and density. Although hunting has frequently been singled out and emphasised as the primary factor contributing to the declining wildlife populations, understanding the connection between hunting and the people is a crucial step towards conservation.

## Graphical abstract

Mizoram's study revealed low ungulate densities and abundance, suggesting illegal hunting as a cause and entailing conservation urgency.

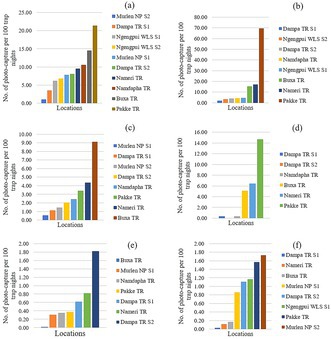

## 1 Introduction

Knowledge of species occurrence, distribution status, and associated threats is a fundamental step for planning and development of conservation strategies (Karanth et al. 2009; Tobler et al. 2008). The absence of baseline knowledge regarding species presence impedes the initiation of any intervention plan. Numerous species, particularly smaller taxa, have gone extinct without being documented, while local extinctions are increasingly prevalent worldwide due to anthropogenic pressures (Régnier et al. 2009; Pereira et al. 2012; Pimm et al. 2014; Ceballos et al. 2015).

Major drivers of biodiversity loss worldwide include overhunting and poaching for meat (Robinson and Bennett 2000; Bennett 2000, 2002; Corlett 2007), illegal wildlife trade (Mills 1993; Robinson 2001; Beissinger 2001; Ripple et al. 2015), habitat degradation due to overexploitation, rapid habitat loss (Sodhi et al. 2004; Schipper et al. 2008; Morrison et al. 2007), logging, and shifting cultivation (Lal and Prajapathi 1990; MacDonald 2001; Raman 2001; Broadbent et al. 2008). Additionally, agricultural expansion, pasturage needs or livestock grazing inside the protected area (Mishra and Rawat 1998; Musavi et al. 2009; Berger et al. 2013; Estrada et al. 2017) further exacerbate biodiversity decline. Mammals are severely threatened all over the world, with 25% of the species at risk of extinction due to anthropogenic factors (Schipper et al. 2008). Over the past century, ungulates have become locally extinct in nearly 52% (range: 25%–81%) of sites, where they were known to occur historically in India (Karanth et al. 2010). Undisturbed ungulate habitats are confined mostly to a network of protected areas and nature reserves that constitute less than 5% of the surviving forests (Karanth et al. 2010).

The state Mizoram, situated in the northeastern region of India is rich in biodiversity due to its geographical position at the Indo‐Myanmar biodiversity hotspot (Barman et al. 2018). The forests of Mizoram play a vital role in sustaining local livelihoods, as a significant portion of the population depends on forest resources. However, despite its high biodiversity, there is scant scientific research and ecological information available on the mammalian fauna of Mizoram, primarily due to the region's rugged topography and limited accessibility (Lalthanzara 2017). Moreover, wildlife conservation authorities in Mizoram have reported the absence of an updated inventory of faunal species within protected areas, and no annual population assessments of predator and prey species are conducted in accordance with the Government of India's guidelines (Lalzidinga 2022).

Although some studies have been conducted on mammals in Mizoram, many lack empirical evidence such as photographic records or museum specimens, as data collection relied on personal experiences, secondary literature, and interviews with local hunters. Additionally, most research efforts are prominently focused on Dampa Tiger Reserve (Dampa TR), the only tiger reserve among the 10 protected areas (PA's) in the state of Mizoram. Consequently, Dampa TR has received comparatively greater scientific attention and protection than the other PA's. Mizoram is also experiencing escalating pressure from illegal wildlife trade, with newly emerging trafficking hubs along its international border with Myanmar, a key player in the illegal wildlife trade in Southeast Asia (Bal and Giordano 2022).

From an ecological perspective, mammals play crucial roles in ecosystems as ecosystem engineers, top predators, herbivores, seed dispersers or pollinators or pest regulators (Jones et al. 1994; Ripple et al. 2014; Ripple et al. 2015; Lacher Jr et al. 2019). They contribute significantly to the structure and functioning of terrestrial ecosystems (Frank et al. 1998; Terborgh et al. 2008; Hobbs 1996; Ripple et al. 2015). In particular, ungulates are essential for sustaining large carnivore populations within a given habitat, as highlighted by Bhattacharya and Sathyakumar (2011). Given the ecological importance of mammals and the existing knowledge gaps in Mizoram, this study aims to generate preliminary data on mammalian diversity in the region. Thus, based on the protected area status, we selected one Tiger Reserve (Dampa TR), one Wildlife Sanctuary, that is, Ngengpui Wildlife Sanctuary (Ngengpui WLS) and one National Park, that is, Murlen National Park (Murlen NP) located in the northwestern, northeastern and southern border of Mizoram respectively.

**FIGURE 1:**
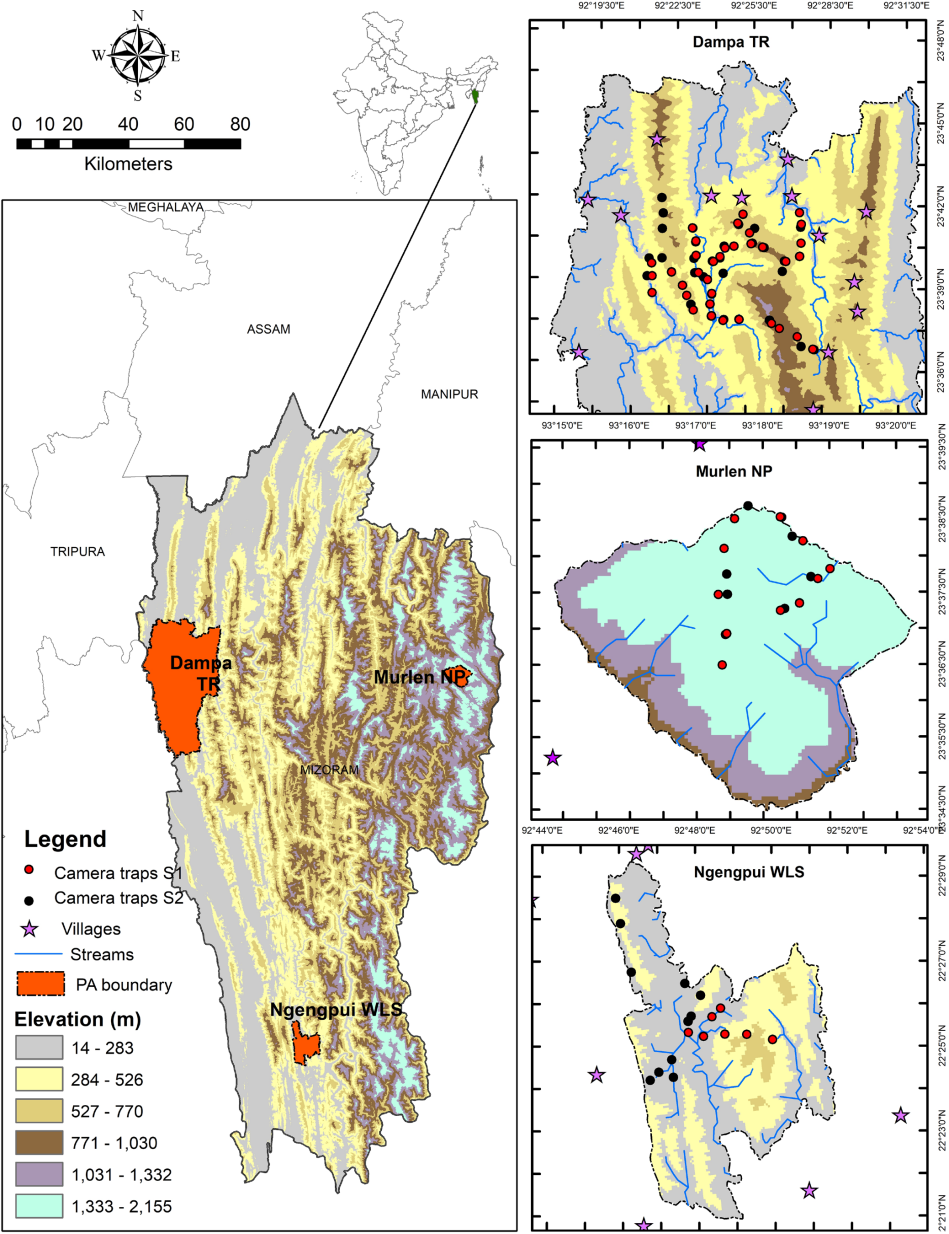
Selected protected areas across Mizoram for the study. Dampa Tiger Reserve (located in the West), Ngengpui Wildlife Sanctuary (located in the South), Murlen National Park (located in the East) with camera trap locations, streams and neighbouring villages.

Dampa TR is located in the Indo‐Myanmar biodiversity hotspot, located in the Lushai Hills, Mamit district at the westernmost boundary of Mizoram (23°20′ N–23°47′ N and 92°15′ E–92°30′ E). It covers an area of 988 Km^2^ of which 500 Km^2^ is the core area (Jhala et al. 2020). The reserve is in contiguity with Bangladesh in the west bordering the Chittagong Hill tract region with elevations ranging from 250 to 1100 m. It receives average annual precipitation of 2150 mm from May to October from the southwestern monsoon. The minimum temperature in winter is 3.5°C and the maximum temperature in the summer is 36°C (Figure 1). Ngengpui WLS is located in the Lawngtlai district in the southwestern part of Mizoram (22°21′18″ N–22°30′01″ N and 91°44′30″ E–92°50′37″ E) and it covers an area of 110 Km^2^. NWLS is enclosed by Chittagong Hills in the west and Chin Hills in the east (Birand and Pawar 2004). The river Ngengpui flows from north to south of the sanctuary and joins the Kolodyne in the south (Birand and Pawar 2004). The sanctuary has an elevation ranging from 200 to 1200 m. It receives annual precipitation of 1700 to 3900 mm and the average temperature is between 8°C and 24°C in winter and 18°C and 32°C in summer (Verma et al. 2013) (Figure 1). Murlen NP is located in the northeastern part of Mizoram in the Champhai district (23°34′28.77″ N–23°38′42.18″ N and 93°14′52.72″ E–93°20′17.94″ E) bordering Myanmar (Kumar et al. 2017). It lies in the Indo‐Myanmar biodiversity hotspot and is close to the Chin Hills (Kumar et al. 2017). The total geographical area covered by the park is 200 Km^2^, of which 100 Km^2^ is notified as the core area (Kumar et al. 2017). The park has an elevation range from 700 to 1800 m and it receives annual precipitation of 2000 to 2500 mm (Sharma, Singh, et al. 2017; Sharma, Kholia, et al. 2017). The minimum and maximum temperatures recorded in the park are 2°C and 35°C respectively (Kumar et al. 2017) (Figure 1).

We hypothesised that the population status of the major predators and ungulates in the PA's of Mizoram will be low due to the increasing anthropogenic constraints and discrepancies in the inventory of the fauna. Therefore, our study objectives were—(a) to prepare an inventory of the mammal species in the region with their relative abundance (b) density of ungulates through a camera trap‐based distance sampling approach rather than the conventional methods (c) to understand the factors influencing ungulates abundance, and (d) to compare the population status of major ungulates and predators of Mizoram with other PA's in India.

## 2 Materials and Methods

### 2.1 Field Surveys

We used stratified systematic sampling and grids of the size of 1 × 1 km^2^ to conduct a camera‐trap survey to assess the abundance and density of mammals. We selected camera‐trap locations based on accessibility, terrain features, and reconnaissance survey. We observed 2 seasons in Mizoram: winter (November–February) and rainy (April–October) (DST, govt. of Mizoram annual report, 2017–2021). In Dampa TR we deployed 40 camera‐traps (February to April 2021 (rainy season); Dampa TR S1) and 29 camera‐traps (November to December 2021 (winter season); Dampa TR S2). In Ngengpui WLS, we deployed 11 camera‐traps (January to February 2022 (winter season); Ngengpui WLS S1) and 5 camera‐traps (December 2022 to January 2023 (winter season); Ngengpui WLS S2). In Murlen NP, we deployed 9 camera‐traps (March to April 2022 (rainy season); Murlen NP S1) and 9 camera‐traps (January to February 2023 (winter season); Murlen NP S2). Trapping effort in a sampling period was the average trap night (functional period of the camera traps when operational) multiplied by the total number of camera‐traps, accounted to 2685 days (Dampa TR S1), 1249 days (Dampa TR S2), 418 days (Ngengpui WLS S1), 205 days (Ngengpui WLS S2), 346 days (Murlen NP S1), and 447 days (Murlen NP S2).

After the completion of each camera‐trapping session, we sorted the camera‐trap pictures to eliminate unusable images (corrupted or highly overexposed, blanks) and corrected the inaccuracies of dates/times on camera settings. To avoid possible false positive detections, we carefully identified the photo‐captured species from a field guide (Menon 2014) and discarded the unclear photographs. We used the camtrapR package (Niedballa et al. 2016) to extract the details of the date and time of each species‐specific capture into a spreadsheet.

### 2.2 Data Analysis

#### 2.2.1 Abundance

We calculated the relative abundance index (RAI) to determine the abundance of the species across the study areas based on photo‐capture rates. To calculate RAI, we considered consecutive photographs of the same species within 30 min as one species occurrence (photographic event) (O'Brien et al. 2003). RAI is calculated as the number of days required for obtaining a photo‐capture of a species (Carbone et al. 2001). We calculated the photo‐capture rate as the number of photographs of a species divided by the number of trap nights per site.

RAI = 100 × (*A*/*N*), *A* = independent photo captures, *N* = trap nights (24 h).

#### 2.2.2 Camera‐Trap Based Distance Sampling (CTDS)

We carried out CTDS (Howe et al. 2017, 2019; Pal et al. 2021) to estimate the density of barking deer, sambar, and wild pig which could not be identified by unique markings. A single motion sensor camera trap was deployed at each location. We kept a time lag of 2 s, burst mode (5 images), and a video of 10 s set between animal captures. Camera traps were mounted at a height of 40 cm–1 m because of the steep and undulated terrain.

We conducted distance sampling based on camera traps by calibrating the area in the field of view (FOV) of the camera traps. We used measuring tapes and a calibration pole (150 cm) to measure the radial distance from the camera traps to the left, right, and center of the camera traps' FOV. We held the calibration pole at every meter from the camera traps, and pictures were taken at every meter through the camera traps for calibration. We compared the camera trap photos of a species to the height of the calibration pole to derive information on the actual height of an individual. For each photo capture moment of an individual, we estimated the radial distance between each individual and the camera trap through a regression equation developed from the field calibration. In this equation, the dependent variable was the ratio of the actual height (shoulder height) of the individual to the height (image size) in the photograph.

The distance data included the animals detected in the camera‐trap locations along with their radial distances (*r*_*i*_), effort and area. The equation for density estimation used by (Cappelle et al. 2019; Howe et al. 2017; Pal et al. 2021) is given as follows

$$\hat{D}=\frac{\sum_{k=1}^{K}n_{k}\;}{{\mathrm{\pi w}}^{2}\;\sum_{k=1}^{K}\mathrm{ek}\;{\hat{P}}_{k}\;}\;x\;1/A$$

where at point *k*, $n_{k}$ is the number of observations, $\mathrm{ek}=\frac{\theta \;T_{k}}{2\mathrm{\pi t}}$ is the temporal effort, $\frac{\theta \;}{2\pi }$ is the fraction of the circle covered by the camera, *θ* degrees (angle covered by the camera's field of view) and $T_{k}$ is the period (in seconds) in which the camera trap was active and t (delay in camera‐trap setting) is the unit of time in seconds in which a finite set of snapshots were obtained. W is the truncation point where any distance beyond this point was discarded, ${\hat{P}}_{k}$ is the estimated probability of obtaining an image of an animal that is within θ. 1/*A* is the availability correction factor.

We used the Distance 7.5 software to analyse the radial distance data through a manual set‐up to model the detection function and hence to estimate *P*_*k*_ (Howe et al. 2017; Pal et al. 2021), where *θ* is assumed to be 45° (0.785). As we have multiple datasets from different sites, firstly the density parameters were analysed by stratum to check model fits and variation in detection probability using the Distance software. Then we pooled the data for each species from different datasets to fit the global detection function curve and estimated the density for both global and stratum resolution. The models used for detection were Uniform function with cosine adjustment, Hazard‐rate function with cosine and hermit polynomial adjustment and Half‐normal function with simple polynomial adjustments. For each analysis the data was transformed into intervals and width was manually specified at 10 m. We selected the best‐fit model based on the value of c‐hat which was within the range of 0.8 to 1.00 and AIC value (Table 1; Table A1).

**TABLE 1:** Best models used for estimating density of each species along with its c‐hat value, AIC and QAIC value.

Species	Model name	No. of parameters	AIC	QAIC	Chi2	c‐hat	EDR
Barking deer	Uniform‐cosine	3	958.22	960.22	0.9039	0.9039	5.6695
Sambar	Uniform‐cosine	2	810.64	812.64	1.6353	0.8176	5.9643
Wild pig	Half normal‐simple polynomial	1	333.47	335.47	3.8501	0.9625	4.678

#### 2.2.3 Relationship With Site Covariates

We were interested in finding out a detailed view of the habitat parameters' effect on the abundance of ungulate species. Herein, we selected 6 site covariates as response variables viz. log‐transformed elevation (m), terrain ruggedness index (Terrain), distance from stream (km) (Dist_strm), log‐transformed ground cover (herb and grass percentage near camera‐trap locations) (GCOV), and log‐transformed number of trees (including bamboos as a measure of denseness of forest near camera‐trap locations). We used the pscl package (Jackman 2020) to carry out the analysis through Hurdle modelling (Cameron and Trivedi 2013). Hurdle models are statistical models used for count data when the data have many zeros. Hurdle models account for the excess zeros and overdispersion. This model structure separately estimates factors influencing (a) the probability of presence (zero hurdle component), and (b) the abundance given presence (count component). Hurdle poisson and Negative binomial models with site covariates were run and the best model was selected based on the Akaike Information Criteria (AIC) and the best fit of the model was confirmed through Vuong's non‐nested test (Table A2).

### 2.3 Temporal Interactions

To understand the relationship between ungulates and major predators with humans, we analysed species temporal interaction. To determine the activity periods of each species, we used the metadata of date and time extracted from camera‐trap photographs. We assumed that the number of camera‐trap records taken at various times was correlated with the daily activity patterns of mammals (Ridout and Linkie 2009). To estimate the overlap coefficient, we used kernel density estimation in the R platform based on the overlap coefficient (Δ), which was defined as the area under the curve that is formed by taking at least two density functions at each time point ranging from 0 (no overlap) to 1 (complete overlap), where 0 implies that the species have no common active period and 1 implies that the activity densities of two species are identical (Schmid and Schmidt 2006).

### 2.4 Comparative Analysis With PA's From the Northeastern Part of India

We collected RAI and density data of 4 tiger reserves from the northeastern part of India: Nameri Tiger Reserve, Pakke Tiger Reserve, Namdapha Tiger Reserve including Buxa Tiger Reserve (Buxa TR) from northeastern West Bengal with similar vegetation and terrain (Table A3) from published literature (2014–2020) (Selvan et al. 2014; Jhala et al. 2020). Then we compared these secondary data with our results from Mizoram to show a comparative assessment of major ungulate and predator populations and density.

## 3 Results

### 3.1 Mammal Assemblage and Abundance

We recorded a total of 27 species of mammals (belonging to 5 orders and 13 families) in our study areas across Mizoram based on camera trap data. In Dampa TR, we recorded 26 species of mammals belonging to 11 families, of the 26 species 14 are of high global conservation significance, categorised as endangered (1), vulnerable (10), and near threatened (3). In Ngengpui WLS, we recorded 17 species of mammals belonging to 10 families. Of the 17 species 10 are of high global conservation significance, categorised as endangered (2), vulnerable (6), and near threatened (2). In Murlen NP, we recorded 14 species of mammals belonging to 8 families. Of the 12 species 5 are of high global conservation significance, categorised as endangered (1), near threatened (1), vulnerable (3). All the categories are based on the IUCN Red List, 2022 (Table 2). No tigers ( *Panthera tigris* ) were photo‐captured during the study.

**TABLE 2:** Number of photo‐captures per 100 trap nights (Relative Abundance Index (RAI)) of mammals in the selected protected areas of Mizoram.

Species	IUCN	*n*	Dampa TR (S1)		*n*	Dampa TR (S2)		*n*	Ngengpui WLS (S1)		*n*	Ngengpui WLS (S2)		*n*	Murlen NP (S1)		*n*	Murlen NP (S2)	
	Status		RAI	SE		RAI	SE		RAI	SE		RAI	SE		RAI	SE		RAI	SE
Carnivora																			
Canidae																			
Dhole	EN	1	0.03	0.32	14	1.11	0.37	5	1.17	1.17	0	—		3	0.86	0.60	8	1.73	1.10
Felidae																			
Golden cat	NT	1	0.03	0.27	3	0.23	0.13	0	—		0	—		0	—		0	—	
Clouded leopard	VU	15	0.61	0.37	23	1.82	0.93	0	—		0	—		1	0.31	0.31	0	—	
Marbled cat	NT	8	0.34	0.12	1	0.07	0.07	3	0.74	0.53	1	0.49	0.49	0	—		8	1.71	1.48
Leopard cat	LC	8	0.29	0.19	16	1.25	0.44	0	—		3	1.46	0.98	0	—		6	1.28	0.55
Herpestidae																			
Crab eating mongoose	LC	5	0.20	0.88	10	0.78	0.36	2	0.47	0.47		—		4	1.14		2	0.42	
Mustelidae																			
Hog badger	VU	17	0.63	0.20	3	0.27	0.20		—		0	—		0	1.67	0.59	0	—	
Yellow throated martin	LC	5	0.17	0.99	8	0.64	0.36		—		1	0.49	0.49	1	0.28	0.28	9	2.12	1.13
Asian small clawed otter	VU	0	—		7	0.54	0.39		0.23	0.23	1	—		0	—		0	—	
Small‐toothed ferret badger	LC	3	0.10	0.96	0	—			—		0	—		0	—		0	—	
Ursidae																			
Malayan sun bear	VU	9	0.35	0.16	26	2.08	1.11	0	—		1	0.49	0.49	0	—		0	—	
Asiatic black bear	VU	5	0.21	0.93	2	0.19	0.19	0	—		0	—		0	—		0	—	
Viviridae																			
Binturong	VU	2	0.07	0.72	0	—		1	0.25	0.25	0	—		0	—		0	—	
Himalayan palm civet	LC	4	0.14	0.67	0	—		0	—		0	—		6	1.75	0.86	0	—	
Common palm civet	LC	35	1.47	0.53	5	0.39	0.20	2	0.47	0.32	2	0.98	0.60	0	—		0	—	
Large Indian civet	LC	2	0.07	0.60	25	1.97	0.82	41	9.63	5.14	3	1.46	0.98	16	4.48	3.25	38	8.50	2.06
Artiodactyla																			
Bovidae																			
Gaur	VU	9	0.33	0.27	0	—		0	—		0	—		0	—		0	—	
Red serow	VU	12	0.51	0.32	6	0.48	0.21	0	—		0	—		0	—		1	0.22	0.22
Cervidae							0.00												
Barking deer	LC	97	3.52	0.69	102	8.04	2.07	26	6.19	3.15	14	6.83	3.96	28	7.85		5	1.06	0.64
Sambar	VU	53	1.92	0.62	50	4.02	1.69	20	4.66	2.31	7	3.41	2.13	0	—		0	—	
Scuidae																			
Wild boar	LC	29	1.11	0.41	22	2.05	1.23	0	—		0	—		2	0.56	0.37	6	1.46	0.59
Perissodactyla																			
Elephantidae																			
Asian elephant	EN	0	—		0	—		7	1.63	1.16	0	—		0	—		0	—	
Primates																			
Cercopithecidae																			
Assamese macaque	NT	3	0.11	0.61	2	0.15	0.11	1	0.25	0.25	0	—		0	—		0	—	
Pig‐tailed macaque	VU	15	0.53	0.18	5	0.39	0.16	1	0.23	0.23	0	—		0	—		0	—	
Rodentia																			
Hystricidae																			
Brush tailed porcupine	LC	26	1.00	0.41	38	2.97	1.28	4	0.98	0.75	1	0.49	0.49	1	0.28	0.28	0	—	
Himalayan crestless porcupine	LC	7	0.27	0.21	26	2.00	1.14	2	0.49	0.33	6	2.93	1.79		—		1	1.50	1.27
Scandentia																			
Northern tree shrew	LC	10	0.36	0.26	2	0.15	0.15		—	—	0	—			—		0	—	

Abbreviations: *n*, np. of photo‐capture; SE, standard error.

Opportunistic encounters during the study recorded evidence of 7 other mammals, which are the endangered hoolock gibbon ( *Hoolock hoolock* ) and phayre's leaf monkey ( *Trachypithecus phayrei* ) including the vulnerable capped langur ( *Trachypithecus pileatus* ), red giant flying squirrel (*Petaurista petaurista*) in Dampa TR. Pallas's squirrel ( *Callosciurus erythraeus* ), hoary‐bellied squirrel ( *Callosciurus pygerythrus* ), and Malayan giant squirrel ( *Ratufa bicolor* ) are from Dampa TR, Ngengpui WLS, and Murlen NP.

Major herbivore species recorded in our survey were barking deer (*Muntiacus vaginalis*; MM), sambar ( *Rusa unicolor* ; RU), wild pig ( *Sus scrofa* ; SC), red serow ( *Capricornis rubidus* ; CR), gaur ( *Bos gaurus* ; BG), and elephant ( *Elephas maximus* ). Major predators were clouded leopard ( *Neofelis nebulosa* ; CL) and dhole ( *Cuon alpinus* ).

The species relative abundance differed among the study areas. The highest RAI of barking deer (8.04 ± 2.07), wild pig (2.05 ± 1.23), red serow (0.51 ± 0.32), and clouded leopard (1.82 ± 0.93) was in Dampa TR. The highest RAI of sambar was in Ngengpui WLS (4.66 ± 2.31) and the highest RAI of Dhole was in Murlen NP (1.73 ± 1.10). Gaur (0.33 ± 0.27) and elephant (1.63 ± 1.16) were only photo‐captured in Dampa TR and Ngengpui WLS respectively. The result showed that among all ungulates, barking deer was the most abundant followed by sambar and wild pig.

### 3.2 Seasonal Variations in RAI

**FIGURE A1:**
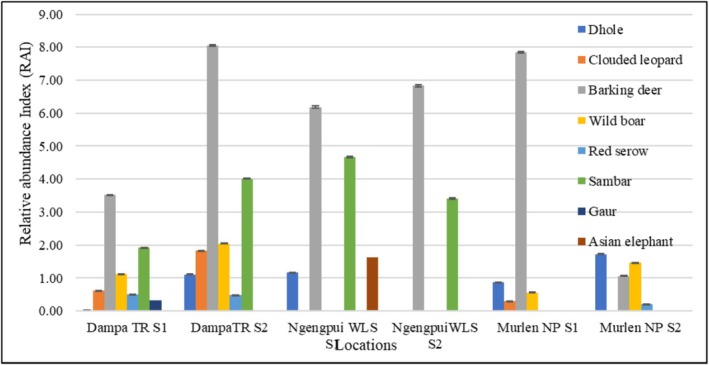
Relative abundance index of major predators and herbivores across three study areas Dampa TR, Ngengpui WLS and Murlen NP in Mizoram. Dampa TR S1 (rainy season), Dampa TR S2 (winter season), Ngengpui WLS S1 and Ngengpui WLS S2 (winter season), Murlen NP S1 (rainy season) and Murlen NP S2 (winter season).

Dampa TR showed the highest relative abundance of barking deer (8.04 ± 2.07), sambar (4.02 ± 0.017), wild pig (2.05 ± 1.23), dhole (1.11 ± 0.37), and clouded leopard (1.82 ± 0.93) during the winter season but no notable seasonal variation in abundance was observed for red serow. In Murlen NP, the highest relative abundance of barking deer (7.85 ± 2.61) and clouded leopard (0.31 ± 0.31) was estimated during the rainy season while, the highest relative abundance of wild pig (1.46 ± 0.59), red serow (0.22 ± 0.22), and dhole (1.73 ± 1.10) was estimated during the winter season (Figure A1). Seasonal variations in Ngengpui WLS could not be assessed due to our inability to undertake a camera‐trapping survey during the spring season.

### 3.3 Density Estimates

**FIGURE A2:**
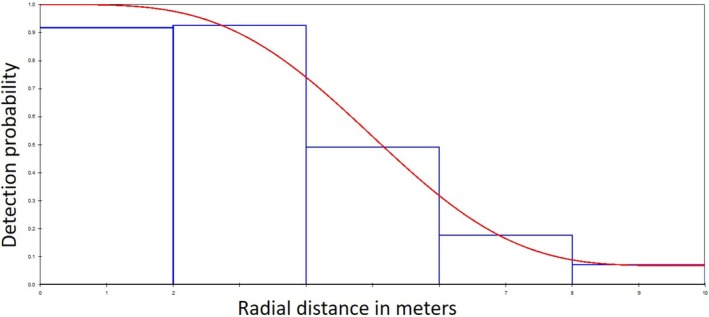
Global detection probability for the best model used for estimating the density of barking deer in Mizoram. The bars show the data distribution, and the line represents the model fit.

**FIGURE A3:**
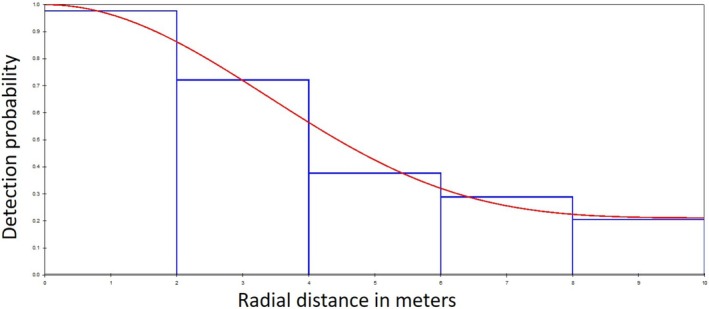
Global detection probability for the best model used for estimating density of sambar in Mizoram. The bars show the data distribution, and the line represents the model fit.

**FIGURE A4:**
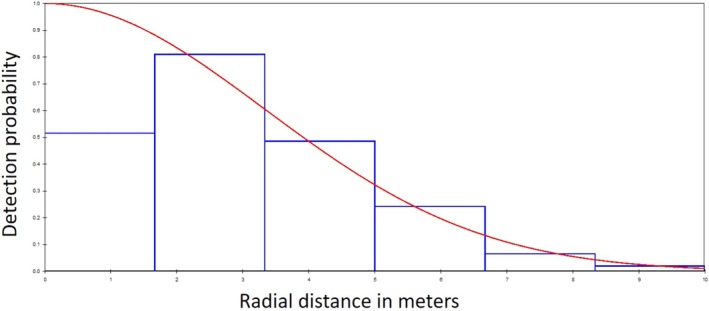
Global detection probability for the best model used for estimating the density of wild pig in Dampa TR. The bars show the data distribution, and the line represents the model fit.

Our study showed that among the ungulates, barking deer has the highest density (Density ± SE) (0.87 ± 0.25) per km^2^, followed by sambar (0.81 ± 0.21) per km^2^ and wild pig (0.45 ± 0.19) per km^2^ (Table 3). Due to the limited photo capture in the camera trap data, we could not carry out density estimation of clouded leopard, dhole, red serow, gaur, and elephants in the study areas. For barking deer and sambar, Uniform models with cosine adjustment were the best fit model for density estimates (Figure A2, Figure A3) while, the Halfnormal model with simple polynomial adjustment was the best fit model for density estimates for wild pig (Figure A4).

**TABLE 3:** Model statistics and parameter estimates of camera‐trap based distance sampling for ungulates in Mizoram in a global density model.

Species	*p* (CV)	EDR (m)	c‐hat	Individual density/km^2^	SE	% CV
Barking deer	0.32 (17.56)	5.67	0.9	0.87	0.25	28.49
Sambar	0.36 (12.46)	5.96	0.82	0.81	0.21	25.91
Wild pig	0.22 (10.72)	4.68	0.96	0.45	0.19	41.29

*Note: p* represents detection probability, EDR represents effective detection radius and c‐hat represents over dispersion factor.

### 3.4 Relationship of Relative Abundance of Ungulates With Site Covariates

Barking deer—Ground cover (log_GCOV) has a strong positive effect on detection probability (Estimate = 1.68, *p* < 0.001), suggesting that denser vegetation improves habitat. Distance to stream has a significant negative effect (Estimate = −0.57, *p* = 0.006), indicating that MM are more likely to occur near water sources. Site‐level variation was marginally significant for NWLS (Estimate = 1.37, *p* = 0.077), suggesting slightly higher detection rates at this site. The rest of the variables are not significant. None of the covariates (log_GCOV, Terrain, Dist_strm, Site) significantly explained variation in mammal counts once presence was established (*p* > 0.1 for all terms). This suggests that, conditional on presence, MM abundance information is limited to obtain a meaningful relationship, or that variation is largely stochastic at the spatial scale examined (Table 4).

**TABLE 4:** Parameter estimates to demonstrate the relationship of relative abundance of ungulates with significant site covariates based on the Hurdle modelling from Zero‐truncated poisson and Zero‐truncated negative binomial regression models. log_GCOV represent log of ground cover, trees represent no. of trees, Terrain represent terrain ruggedness index, Dist_strm represent distance to stream and sites represent the different survey sites.

Species	Covariates	Estimate	Std. error	*z*	*p*
Barking deer	*Count model coefficients (truncated negative‐binomial with log link)*				
	(Intercept)	1.86	0.96	1.93	0.054
	log_GCOV	0.32	0.29	1.10	0.270
	Terrain	−1.14	1.51	−0.76	0.449
	Dist_strm	−0.07	0.15	−0.48	0.631
	SiteMNP	0.51	0.48	1.05	0.293
	SiteNWLS	0.73	0.46	1.57	0.116
	Log(theta)	0.03	0.31	0.11	0.912
	*Zero hurdle model coefficients (binomial with logit link)*				
	(Intercept)	−1.07	1.57	−0.68	0.498
	log_GCOV	**1.68**	0.48	3.51	**0.000**
	Terrain	−0.88	2.54	−0.35	0.728
	Dist_strm	**−0.57**	0.21	−2.74	**0.006**
	SiteMNP	0.78	0.72	1.08	0.281
	SiteNWLS	**1.37**	0.78	1.77	**0.077**
Red serow	*Count model coefficients (truncated poisson with log link)*				
	(Intercept)	−0.38	6.17	−0.06	0.951
	log.elevation	−0.04	1.01	−0.04	0.967
	Terrain	**7.79**	3.24	2.40	**0.016**
	logtree	**−1.85**	0.77	−2.42	**0.016**
	*Zero hurdle model coefficients (binomial with logit link)*				
	(Intercept)	−31.79	11.87	−2.68	0.007
	log.elevation	**4.42**	1.68	2.64	**0.008**
	Terrain	0.98	4.80	0.20	0.839
	logtree	0.92	0.91	1.01	0.315
Sambar	*Count model coefficients (truncated negative‐binomial with log link)*				
	(Intercept)	8.00	4.09	1.95	0.051
	log_elevation	**−1.11**	0.61	−1.83	**0.067**
	log_GCOV	−0.15	0.15	−1.02	0.310
	log_trees	0.27	0.17	1.59	0.113

	SiteNWLS	−0.35	0.85	−0.41	0.682
	Log(theta)	−0.18	0.50	−0.35	0.726
	*Zero hurdle model coefficients (binomial with logit link)*				
	(Intercept)	10.02	4.47	2.24	0.025
	log_elevation	**−1.61**	0.63	−2.55	**0.011**
	log_GCOV	0.09	0.19	0.46	0.644
	log_trees	−0.26	0.20	−1.29	0.198
SiteNWLS	**−1.53**	0.92	−1.67	**0.095**
Wild pig	*Count model coefficients (truncated negative‐binomial with log link)*				
	(Intercept)	−18.62	8.06	−2.31	0.021
	log_elevation	**3.42**	1.26	2.72	**0.007**
	log_GCOV	−0.05	0.27	−0.20	0.842
	Terrain	−3.65	5.38	−0.68	0.497
	SiteMNP	**−4.66**	1.97	−2.36	**0.018**
	Log(theta)	−0.53	0.81	−0.66	0.512
	*Zero hurdle model coefficients (binomial with logit link)*				
	(Intercept)	−7.49	4.08	−1.84	0.066
	log_elevation	**1.15**	0.63	1.84	**0.066**
	log_GCOV	0.08	0.17	0.46	0.643
	Terrain	−1.51	2.69	−0.56	0.576
	SiteMNP	−1.03	0.99	−1.04	0.300

*Note:* Bold are statisticaly significant (*p* < 0.1).

Sambar—Elevation has a negative influence on the presence (Estimate = −1.61, *p* = 0.011) and abundance (Estimate = −1.11, *p* = 0.067) of sambar indicating elevation as a determiner of sambar presence and abundance while there is a significant site‐wise (Ngengpui WLS). Difference in presence (Estimate = −1.53, *p* = 0.011) but not in abundance response (Table 4).

Red serow—Presence of red serow increases with elevation (Estimate = 4.42, *p* = 0.008) while abundance is negatively influenced by the number of trees (Estimate = −1.85, *p* = 0.016) and terrain ruggedness has a significant positive effect (Estimate = 7.79, *p* = 0.016) on the red serow abundance. However, the sample size was small as it was only recorded in Dampa TR (Table 4).

Wild pig—The elevation gradient has a significant positive effect on presence (Estimate = 1.15, *p* = 0.066) and abundance (Estimate = 3.42, *p* = 0.007) of wild pig and there is site‐wise response to abundance (Estimate = −4.66, *p* = 0.018) suggesting slightly lesser detection rates at this site (Murlen NP) (Table 4).

Models could not be computed for gaur and elephants due to low capture of species.

### 3.5 Temporal Interactions

**FIGURE 2:**
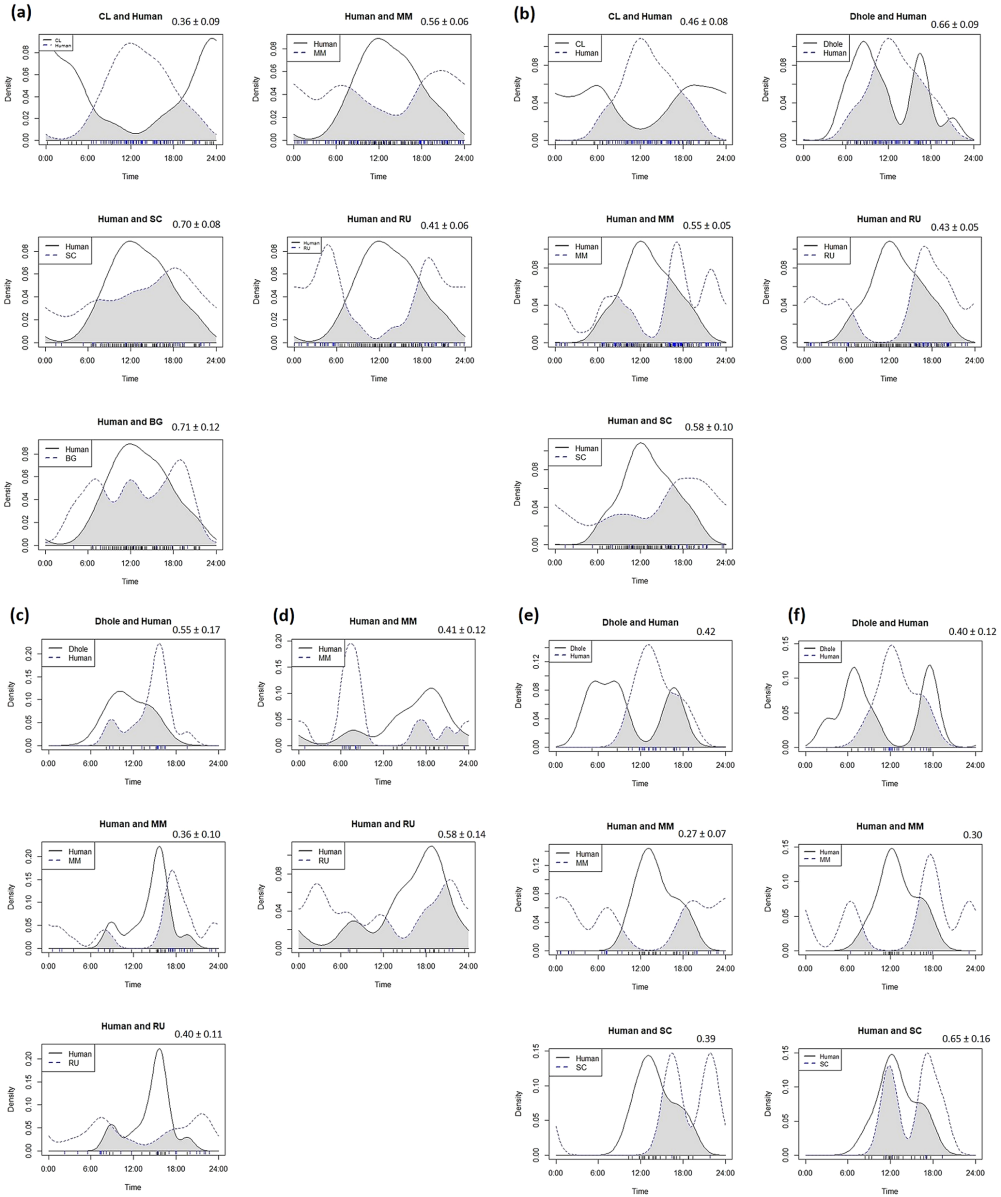
Overlap coefficients of activity patterns ± SD of major predators and ungulates with human in (a) Dampa TR S1, (b) Dampa TR S2, (c) Ngengpui WLS S1, (d) Ngengpui WLS S2, (e) Murlen NP S1 AND (f) Murlen NP S2. Here, clouded leopard (CL), dhole, barking deer (MM), sambar (RU), wild pig (SC).

Throughout the camera‐trapping session in all the study areas ungulate and predator species had different activity periods relative to humans, meaning they had low temporal overlap with humans except for wild pig and gaur in Dampa TR during the rainy season (Figure 2).

### 3.6 Comparative Analysis With PA's From the Northeastern Part of India

**FIGURE 3:**
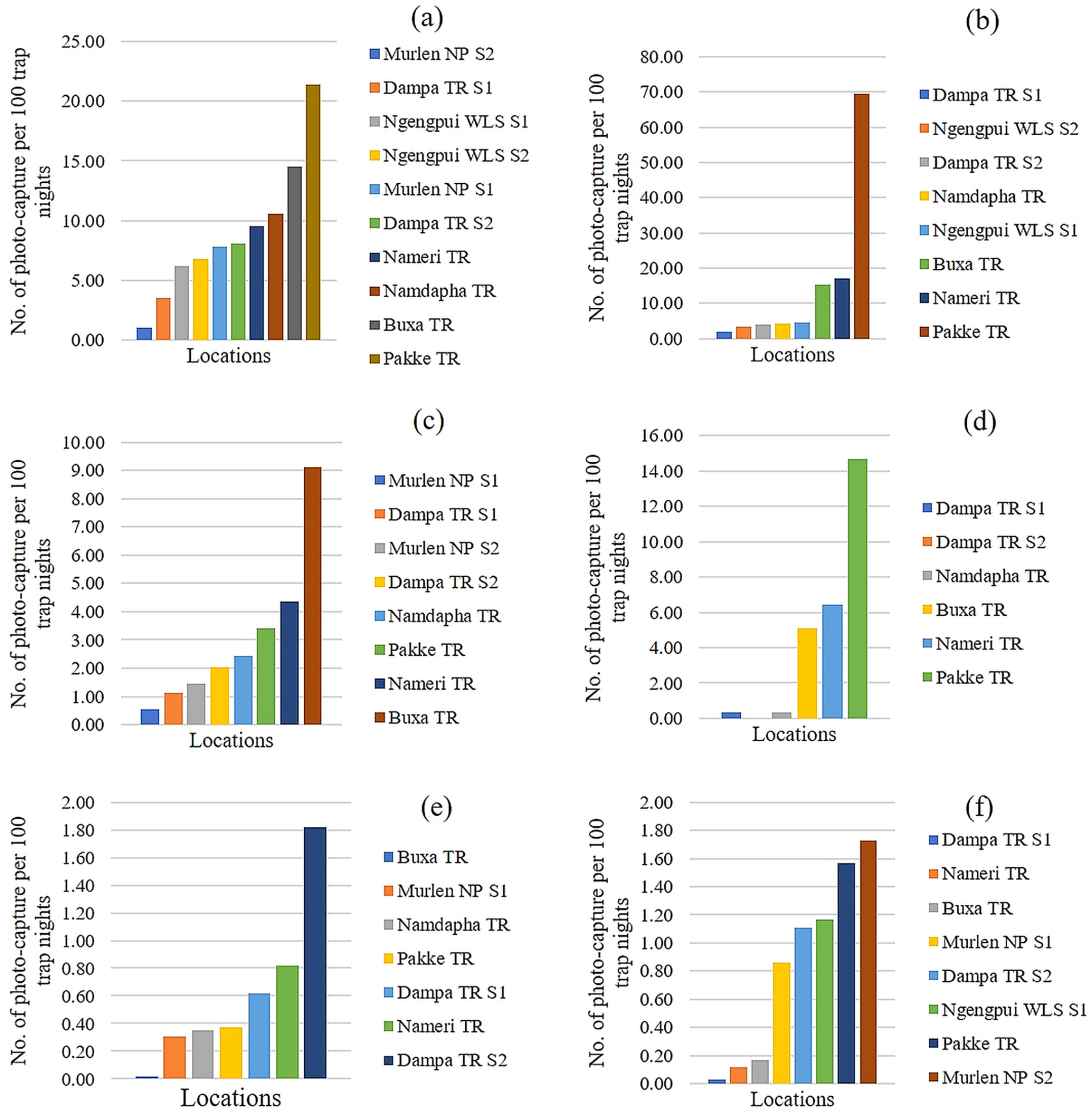
Comparative assessment of relative abundance index (RAI) and density of ungulates and relative abundance index (RAI) of major carnivores from PA's of Mizoram and selected tiger reserves from the northeastern part of India including Buxa TR with similar vegetation type and terrain. (a) Barking deer, (b) sambar, (c) wild pig, (d) gaur, (e) clouded leopard and (f) dhole.

**FIGURE 4:**
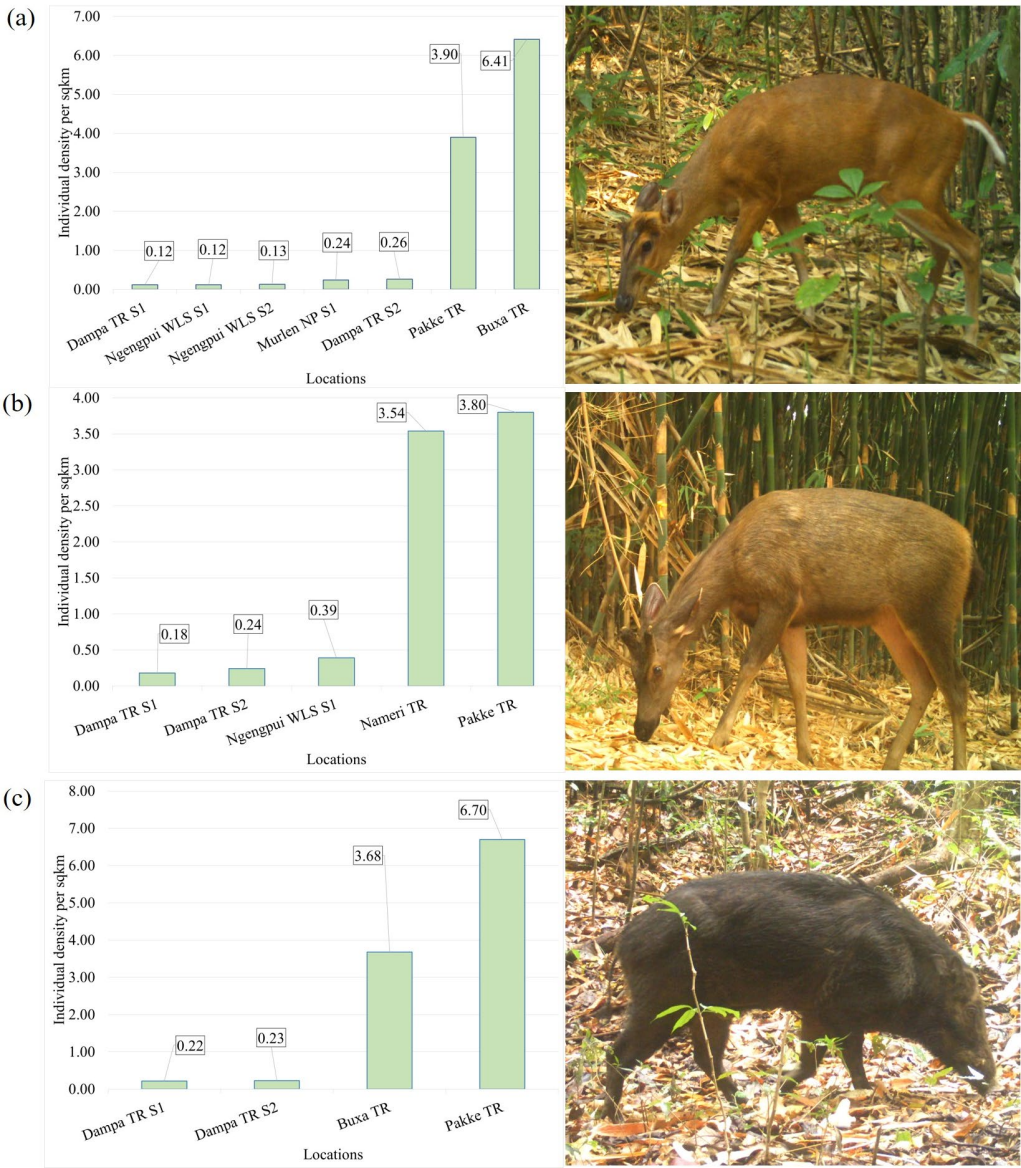
Comparative assessment of density estimates of ungulates per km^2^ from PA's of Mizoram and selected tiger reserves from the northeastern part of India including Buxa TR. (a) Barking deer, (b) sambar and (c) wild pig.

Comparative analysis to assess the population dynamics of the major ungulate species such as barking deer, sambar, wild pig, and gaur from PA's in Mizoram exhibited lower RAI and density than other PA's of northeast India (Figures 3 and 4). Among the studies, the relative abundance of barking deer, sambar, and gaur was highest in Pakke TR and wild pig in Buxa TR. Buxa TR also recorded the highest density of barking deer (6.41 ± 1.16 individuals per km^2^), whereas Pakke TR exhibited the highest density for sambar (3.80 ± 0.50 individuals per km^2^) and wild pig (6.70 ± 1.20 individuals per km^2^). The relative abundance of major predators i.e., clouded leopard and dhole was found highest in PA's of Mizoram relative to other PA's (Figure 3) (Table A4).

## 4 Discussion

This study provides information on the species presence based on photographic evidence in the PA's of Mizoram along with the status of the ungulate population through camera‐trap based distance sampling and the abundance of major predators. The CTDS method appears to be an effective way to assess the status of the wild population in our study areas as many previous studies in similar habitats highlighted the difficulties and inability to assess the densities through standard practices based on visual encounters (Eisenberg and Seidensticker 1976; Karanth et al. 2004; Datta et al. 2008). Through camera trapping we reported the presence of 15 species of high global conservation significance from Mizoram such as endangered dholes, rare species like clouded leopard and Malayan sun bear, Asian elephant, and red serow.

### 4.1 Species‐Covariates Relationship From the PA's

The population demography and dynamics of ungulates are significantly influenced by changing habitat conditions (Owen‐Smith and Mills 2006), as well as by prevailing weather and climatic variables, which indirectly affect the forage quality (Sæther 1997; Post and Stenseth 1999; Hone and Clutton‐Brock 2007) and temporal availability of food resources (Illius and O'Connor 2000). In forested habitats, fruits, flowers, leaves, and bamboo shoots are available in the pre‐monsoon season (Liese and Köhl 2015; Awasthi et al. 2016). Grasses grow during the monsoon or rainy season, mature during winter and can persist till early summer or pre‐monsoon season. Although the peak abundance of forage typically coincides with the rainy season, certain species such as *Terminalia chebula* and *Elaeocarpus* spp. were recorded during the winter season in the present study, thereby providing alternative food sources during periods of relative forage scarcity. A high abundance of sambar and wild pig was estimated during the winter season in our study, which very well coincides with the forage availability and their peak rut season (Schaller 1967; Santiapillai and Chambers 1980). The abundance of barking deer which breed all year round, and serow being a generalist feeder (Giri et al. 2011) were not affected by the seasonal shifts. The availability of forage, in conjunction with herb density and overall food quality, has been identified as a critical determinant of habitat selection in barking deer ( *Muntiacus muntjak* ) (Teng et al. 2004; Kushwaha et al. 2004; Gurung 2022). Furthermore, Barrette (1977) noted that barking deer are often associated with water bodies, likely to fulfill their water requirements. In concordance with these findings, the present study observed a strong influence of ground vegetation cover on the spatial occurrence of barking deer. Moreover, the proximity to streams emerged as a significant predictor, negatively correlating with the presence of the species within the study landscape, suggesting that access to water sources may play an important role in habitat preference and usage.

The occurrence of the red serow ( *Capricornis rubidus* ) in Mizoram exhibits a significant positive correlation with both elevation and terrain ruggedness, while showing a negative association with the richness of trees across survey areas. This pattern suggests a preference for steep and elevated habitats that are less forested. Previous studies have documented the presence of red serows in mountainous regions and tropical forests characterised by rugged terrain in northeastern India (Duckworth and Than 2008; Erard 2017). Mountainous and rugged landscapes often exhibit a decline in tree density with increasing elevation and topographic complexity (Acharya et al. 2011; Rawat et al. 2021; Yadav et al. 2025). This aligns with our findings, where a significant decrease in the number of trees was recorded with increasing elevation (Table A5). Although specific ecological studies on *Capricornis rubidus* remain limited, the species is often presumed to occupy habitats similar to those of *Capricornis sumatraensis* , given their morphological and ecological similarities (Duckworth and Than 2008; Erard 2017).

On the contrary, our study revealed a significant negative correlation between sambar occurrence and elevation. Although sambar is a highly adaptable ungulate (Schaller 1967), they are known to prefer well‐watered, moist deciduous hilly terrains (Kumar 2000). Previous studies have documented sambar presence predominantly in areas with high tree and herb density (Kushwaha et al. 2004) and a preference for tree canopy cover (Sankar and Acharya 2004). Our findings indicate a decline in the number of trees and an increase in distance to water sources, such as streams, with increasing elevation (Table A5). These habitat characteristics may underlie the observed negative association between sambar presence and abundance with elevations in our study area.

Wild pigs are highly adaptable and generalist species capable of inhabiting a wide range of habitat (Prater and Barruel 1971; Mayer 2009). In the present study, although all surveyed sites exhibited similar vegetation types and elevation gradients, significant variation was observed in the frequency of wild pig detections across sites and has a positive correlation with elevation. The majority of photo‐capture records originated from Dampa TR, with few from Murlen NP and none from Ngengpui WLS, which might be due to human disturbances. While elevation alone may not solely determine the species' distribution, factors such as survival instincts, predatory pressure and human activities can push the species to higher elevation and slope (Paudel and Kindlmann 2012). In the Northeastern India region, wild meat is often regarded as a luxury commodity or a “pure” form of meat, preferred over domestic meat (Aiyadurai 2011). Among the various hunted species, wild pigs are notably favoured by local communities in Northeast India and widely preferred as wild meat (D'Cruze et al. 2018; Lyngdoh et al. 2023; Longchar et al. 2025).

### 4.2 Comparative Analysis With PA's Across India

We have found that Mizoram had the highest relative abundance of clouded leopards and dholes relative to other PA's of northeast India, which is comparable to previous studies in Dampa TR; an important refuge for dhole (Singh et al. 2020). Conversely, a low relative abundance of ungulate species was recorded in Mizoram relative to other PA's. Our findings demonstrated that barking deer was the most abundant ungulate among all, potentially attributed to its resilience to hunting pressure compared to larger ungulates in areas of frequent hunting (Datta et al. 2008). Unlike sambar, barking deer are known to persist at reduced densities with hunting pressure (Pattanavibool and Dearden 2002; Tungittiplakorn and Dearden 2002) and they can recover relatively rapidly from low population levels (Steinmetz et al. 2010). Endangered species like dholes, one of the major predators in Mizoram prefer medium‐sized prey such as barking deer (Aiyadurai and Varma 2003), wild pig (Gopi et al. 2010) followed by sambar (Gopi et al. 2010; Karanth and Sunquist 1995; Johnsingh 1983) and gaur (Gopi et al. 2010). Consequently, the declining populations of ungulate species, which are the preferred prey of dholes, will probably impact the population dynamics of dholes in the long run.

The absence of tigers in Dampa Tiger Reserve, reflects the grim reality of their declining numbers, shrinking habitats and declining prey species in Mizoram. Despite its status as a tiger reserve, recent surveys have failed to record any presence of the majestic cat, highlighting the alarming rate at which their geographical range is diminishing. According to the forest cover assessment report 2019, Mizoram holds the highest percentage of forest cover under a highly fire‐prone class in India.

Drivers behind low densities in PA's of Mizoram are believed to be a combined effect of anthropogenic activities and hunting as local communities in and around the PA's of Mizoram rely on wildlife resources. The biggest contributing factor to the demise of the largest terrestrial herbivores is probably widespread overhunting for meat across most of the developing countries (Milner‐Gulland and Bennett 2003; Craigie et al. 2010; Lindsey et al. 2013; Brashares et al. 2014). Southeast Asia has emerged as a prominent epicentre for the wildlife trade (Sodhi et al. 2004), thereby exposing ungulate species to the risk of extinction. The wanton hunting of these species has escalated over the past few decades to meet the demands of local and regional markets, where their meat and body parts are commercially sought after (Srikosamatara and Suteethorn 1994; Nooren and Claridge 2001; Sodhi et al. 2004; Corlett 2007). In Southeast Asia, Myanmar has a significant role in the illegal wildlife trade (Bal and Giordano 2022). Nijman and Shepherd (2015) observed over 2000 cat parts, including 1428 skins, representing a minimum of 1669 individual cats where the clouded leopard (482 individuals) was the most common species in trade. There is a massive platform of illegal wildlife trade in Mizoram with Champhai district as the hotspot due to its international boundaries with Myanmar and Bangladesh (Bal et al. 2023). According to a study by Solanki and Lalchhandama (2016), around 525 wild animals are hunted by local people annually in Dampa TR over 30 years. During the study period, we encountered several poaching signs such as animal remains, signs of fishing, old machans for hunting, gunpowder remains, the incidences of gunshot noise and chainsaw noise deep inside the forest. Hunting is an age‐old tradition for the indigenous tribes of Mizoram primarily for meat and secondarily for trophies. Hence, they cannot easily abandon this practice (Gupta and Sharma 2005; Lalzidinga 2022). High demands for the skin and bones of tigers and leopards, scales of pangolin, horns of gaur, sambar, and serow, and bear bile encourage the villagers to commercial hunting while, hunting for commercial purposes puts a huge toll on the wildlife (Lalzidinga 2022). Bal et al. 2023 mentioned in their study that 723 animals of numerous species were seized from January 2021 to October 2022 from the Mizoram–Myanmar border. Murlen NP situated at the Indo‐Myanmar international border is rich in biodiversity but its biodiversity has suffered immensely in the past few decades due to constant anthropogenic pressure such as hunting and jhuming (shifting cultivation) (Gupta and Sharma 2005). The presence of a large number of guns and suspected illegal entry into the park from the people of Myanmar have further contributed to the cause (Gupta and Sharma 2005). Hunting with guns is the most common method of killing wild animals by the villagers residing near the PA's becausehaving a gun is a profitable deal for gun owners as they still practice the old tradition of giving animal tax (in the form of meat) to the owner of the gun upon borrowing it for hunting, which encourages the villagers to own a gun (Lalzidinga 2022). By 2018, 21,189 persons were registered with licensed arms in Mizoram (Lalzidinga 2022). According to No. F.12013/5/2020‐CWLW (dated 9th February 2022), the presence of 375 licensed arms/guns was affirmed in the fringe villages of the PA's viz., Dampa TR, Lengteng WS (Lengteng Wildlife Sanctuary), MNP, and TWS (Tawi Wildlife Sanctuary). From the year 2006 to 2019, 393 wildlife offence cases were recorded in Mizoram (Statistical Handbook 2011, EFCC, Mizoram and office of CCF, Mizoram, EFCC). The annual rate of occurrence of wildlife offence cases during this period was 28. From the year 2008 to 2019, there were a total of 197 wildlife offence cases from Dampa TR alone (office of FD, Dampa Tiger Reserve, Mizoram and office of CCF, Mizoram, EFCC). Most of the wildlife offence cases occur in the fringe villages near PA's and villagers are still involved in hunting and killing wild animals in and around the PA's of Mizoram.

Hunting has often been singled out and highlighted as the major reason for the decline of wildlife populations in the state (Pawar and Birand 2001; Mishra et al. 2006; Aiyadurai 2011), However, understanding the link between hunting and the people involved, and addressing the social, historical, political, and economic contexts is an effective step towards conservation. Assessment of species abundance, diversity and distribution is crucial for the effective management of biodiversity in a region. The occurrence of mammal species with global conservation significance, low abundance and density of the ungulates from the PA's of Mizoram portrays the urgency of a conservation plan in this region. In addition to this, to Aiyadurai (2007) stated that long‐term conservation goals depend on local people's attitudes and perceptions of wildlife (Choudhury et al. 2019; Ebua et al. 2011; Htun et al. 2012; Newmark et al. 1993), especially in a region where hunting by tribal communities has wider cultural importance than just economic benefits.

The relationship between people and wildlife is complex, and solving issues around it takes time. In areas where hunting is a traditional practice and people depend heavily on forests, it can be difficult to gain local support for protecting wildlife and maintaining the long‐term integrity of the protected areas. However, management efforts can help improve people's understanding and awareness of wildlife. Organising workshops or training sessions on local wildlife and basic animal behaviour is important to promote human‐wildlife coexistence. While traditional beliefs can support conservation, modern, unsustainable practices especially among youth who are disconnected from their cultural roots often work against it. This highlights the need to incorporate subjects on the importance of biodiversity and conservation into the curriculum of local educational institutions to foster awareness and a sense of responsibility from an early age.

## Author Contributions

**Akangkshya Priya Gogoi:** conceptualization (equal), formal analysis (lead), methodology (equal), writing – original draft (lead). **Lallianpuii Kawlni:** conceptualization (equal), funding acquisition (lead), project administration (equal), resources (equal), supervision (equal), writing – review and editing (equal). **Joonu Chakma:** conceptualization (supporting), formal analysis (supporting), methodology (supporting). **Vishnupriya Kolipakam:** conceptualization (equal), investigation (equal), supervision (supporting), writing – review and editing (equal). **Qamar Qureshi:** conceptualization (equal), formal analysis (supporting), investigation (equal), methodology (supporting), project administration (equal), supervision (equal), writing – review and editing (equal).

## Conflicts of Interest

The authors declare no conflicts of interest.

## Data Availability

The data that support the findings of this study are openly available in https://zenodo.org at https://zenodo.org/records/16376697.

## Appendix A

**TABLE A1:** All models used for estimating the density of each species along with its c‐hat value and AIC value for selection of the best fit model.

Species	Model name	No. of parameters	AIC	QAIC	Chi2	df	c‐hat	*p*	EDR
Barking deer	**Uniform‐cosine***	**3**	**958.22**	**960.22**	**0.9039**	**1**	**0.9039**	**0.34175**	**5.6695**
	Half normal‐hermit polynomial	1	957.15	959.15	3.7328	3	1.2443	0.2918	5.1664
	Half normal‐simple polynomial	1	957.15	959.15	3.7328	3	1.2443	0.2918	5.1664
	Hazard rate‐cosine	3	957.39	959.39	0.0789	1	0.0789	0.77878	5.8108
Sambar	**Uniform‐cosine***	**2**	**810.64**	**812.64**	**1.6353**	**2**	**0.8176**	**0.44148**	**5.9643**
	Half normal‐hermit polynomial	1	814.86	816.86	7.8394	3	2.6131	0.04945	6.8046
	Half normal‐simple polynomial	3	811.2	813.2	0.1952	1	0.1952	0.6586	5.5933
	Hazard rate‐cosine	2	809.89	811.89	0.8753	2	0.4376	0.64556	5.7067
Wild pig	Uniform‐cosine	2	334.64	336.64	3.1498	3	1.0499	0.36911	4.8376
	Half normal‐hermit polynomial	2	333.39	335.39	2.166	3	0.722	0.53867	5.3242
	**Half normal‐simple polynomial***	**1**	**333.47**	**335.47**	**3.8501**	**4**	**0.9625**	**0.42667**	**4.678**
	Hazard rate‐cosine	2	333.77	335.77	2.6399	3	0.88	0.45054	5.6417

*Note:* The models with the asterisk and highlighted in bold letters are the best models.

**TABLE A2:** Log likelihood (logLik), AIC and weight of Hurdle regression models used to fit relative abundance of barking deer, sambar, wild pig and red serow along with the Vuong *z*‐values for selection of best fit model.

Species	Model	Model_parameters	logLik	AIC	Weight
Barking deer	Poisson	log_GCOV + Terrain + Dist_strm + Site | log_GCOV + Terrain + Dist_strm + Site	−387.80	799.61	0.00
	Negative binomial	log_GCOV + Terrain + Dist_strm + Site | log_GCOV + Terrain + Dist_strm + Site	−255.20	536.31	1.00
Red serow	Poisson	log.elevation + Terrain + logtree	−33.05	82.10	0.86
	Negative binomial	log.elevation	−37.86	85.72	0.14
Sambar	Poisson	log_elevation + log_GCOV + Terrain + log_trees + Site | log_elevation + log_GCOV + Terrain + log_trees + Site	−209.20	442.46	0.00
	Negative binomial	log_elevation + log_GCOV + log_trees + Site | log_elevation + log_GCOV + log_trees + Site	−151.50	325.00	1.00
Wild pig	Poisson	log_elevation + log_GCOV + Terrain + Site | log_elevation + log_GCOV + Terrain + Site	148.8	317.59	0.00
	Negative binomial	log_elevation + log_GCOV + Terrain + Site | log_elevation + log_GCOV + Terrain + Site	112.8	247.58	1.000

Vuong's non‐nested test for best fit					
	Vuong *z*‐statistic	Model comparison	*z*	*p*	
Barking deer	Raw	Negative binomial > Poisson	−2.41	0.008	
	AIC‐corrected	Negative binomial > Poisson	−2.41	0.008	
	BIC‐corrected	Negative binomial > Poisson	−2.41	0.008	
Red serow	Raw	Poisson > Negative binomial	1.73	0.042	
	AIC‐corrected	Poisson > negative Binomial	0.29	0.386	
	BIC‐corrected	Negative binomial > Poisson	−1.32	0.094	
Sambar	Raw	Negative binomial > Poisson	−2.11	0.017	
	AIC‐corrected	Negative binomial > Poisson	−2.18	0.015	
	BIC‐corrected	Negative binomial > Poisson	−2.27	0.012	
Wild pig	Raw	Negative binomial > Poisson	−1.51	0.065	
	AIC‐corrected	Negative binomial > Poisson	−1.51	0.065	
	BIC‐corrected	Negative binomial > Poisson	−1.51	0.065	

*Note:* log_GCOV represents log of ground cover, trees represent the no. of trees, Terrain represents terrain ruggedness index, Dist_strm represents distance to stream and sites represent the different survey sites. + is additive effect and | separates count model and binomial parts of the model.

**TABLE A3:** List of protected areas from the northeastern part of India including Buxa Tiger Reserve with their habitat characteristics.

Protected areas	Location	Area (km^2^)	Elevation (m)	Rainfall (mm)	Dominant vegetation
Dampa Tiger Reserve	Mizoram	988	250–1100	2150	Tropical semi‐evergreen forest, bamboo brakes and mixed deciduous forests
Nnengpui Wildlife Sanctuary	Mizoram	110	200–1200	1700–3900	Tropical moist evergreen
Murlen National Park	Mizoram		700–1800	2000–2500	Sub‐tropical semi evergreen and semi‐montane type
Nameri Tiger Reserve	Assam	464	100–275	2141.54	Eastern alluvial secondary semi‐evergreen forest, low alluvial savannah woodland, eastern Dillenia swamp forest and wet bamboo forest with cane brakes
Pakke Tiger Reserve	Arunachal Pradesh	1198.45	150–2000	2500	Assam valley tropical evergreen, tropical semi‐evergreen sub‐tropical broadleaved forests
Namdapha Tiger Reserve	Arunachal Pradesh	2052.82	200–4571	200–4300	Sub‐tropical broadleaved rainforests, subtropical pine forest temperate broadleaved forest and alpine meadows
Buxa Tiger Reserve	West Bengal	757.9	60–1904	4100	Moist tropical forest, semi‐evergreen forest, evergreen forest, wet mixed forest, hill forest, moist mixed/dry mixed forest, sal forest, savannah forest and riverine forest

**TABLE A4:** Comparative assessment of relative abundance index (RAI) and density of ungulates and relative abundance index (RAI) of major carnivores from PA's of Mizoram and selected tiger reserves from the northeastern part of India including Buxa TR.

Locations	RAI	Density (SE)/km^2^	RAI	Density (SE)/km^2^	RAI	Density (SE)/km^2^	RAI
Ungulates	Barking deer		Sambar		Wild pig		Gaur
Dampa TR S1	3.52	0.12 (0.02)	1.92	0.18 (0.06)	1.11	0.22 (0.12)	0.33
Dampa TR S2	8.04	0.26 (0.08)	4.02	0.24 (0.08)	2.05	0.23 (0.14)	0.00
Ngengpui WLS S1	6.19	0.12 (0.08)	4.66	0.39 (0.17)	—	—	—
Ngengpui WLS S2	6.83	0.13 (0.06)	3.41	—	—	—	—
Murlen NP S1	7.85	0.24 (0.16)	—	—	5.56	—	—
Murlen NP S2	1.06	—	—	—	1.46	—	—
Nameri TR	9.53	—	16.95	3.54 (1.11)	4.35	—	6.45
Pakke TR	21.40	3.9 (0.60)	69.36	3.80 (0.50)	3.43	6.70 (1.20)	14.69
Namdapha TR	10.55		4.17	—	2.43	—	0.35
Buxa TR	14.56	6.41 (1.16)	15.25	—	9.12	3.68 (1.26)	5.08
Carnivores	Clouded leopard		Dhole				
Dampa TR S1	0.61	—	0.03	—	—	—	—
Dampa TR S2	1.82	—	1.11	—	—	—	—
Ngengpui WLS S1	—	—	1.17	—	—	—	—
Ngengpui WLS S2	—	—	—	—	—	—	—
Murlen NP S1	0.31	—	0.86	—	—	—	—
Murlen NP S2		—	1.73	—	—	—	—
Nameri TR	0.82	—	0.12	—	—	—	—
Pakke TR	0.37	—	1.57	—	—	—	—
Namdapha TR	0.35	—		—	—	—	—
Buxa TR	0.02	—	0.17	—	—	—	—

**TABLE A5:** Correlation between elevation and habitat covariates with *p*‐values.

log_elevation	Correlation coefficient	*p*
Trees	−0.26192	0.015458
log_trees	−0.26171	0.015544
Distance to stream	0.234	0.031128
log_ground cover	0.13121	0.23132
